# Combined opioid free and loco-regional anaesthesia enhances the quality of recovery in sleeve gastrectomy done under ERAS protocol: a randomized controlled trial

**DOI:** 10.1186/s12871-021-01561-w

**Published:** 2022-01-21

**Authors:** Mohamed Ibrahim, Ali M. Elnabtity, Ahmed Hegab, Omar A. Alnujaidi, Osama El Sanea

**Affiliations:** 1 Department of Anesthesiology, Faculty of Medicine, Zagazig University, Zagazig, Egypt; 2 Al Mashfa Medical Center, Arraka Shamalia Dist., Khalid Ibn Elwaleed Street, Al Khobar, Kingdom of Saudi Arabia

**Keywords:** Opioid free anaesthesia, Multimodal analgesia, Quality of recovery, Opioid consumption

## Abstract

**Background:**

It is debatable whether opioid-free anaesthesia (OFA) is better suited than multimodal analgesia (MMA) to achieve the goals of enhanced recovery after surgery (ERAS) in patients undergoing laparoscopic sleeve gastrectomy.

**Methods:**

In all patients, anaesthesia was conducted with an i.v. induction with propofol (2 mg. kg-1), myorelaxation with cisatracurium (0.15 mg.kg-1), in addition to an ultrasound-guided bilateral oblique subcostal transverse abdominis plane block. In addition, patients in the OFA group (*n* = 51) received i.v. dexmedetomidine 0.1 μg.kg-1 and ketamine (0.5 mg. kg-1) at induction, then dexmedetomidine 0.5 μg. kg-1.h-1, ketamine 0.5 mg.kg-1.h-1, and lidocaine 1 mg. kg-1.h-1 for maintenance, while patients in the MMA group (*n* = 52) had only i.v. fentanyl (1 μg. kg-1) at induction. The primary outcome was the quality of recovery assessed by QoR-40, at the 6th and the 24th postoperative hour. Secondary outcomes were postoperative opioid consumption, time to ambulate, time to tolerate oral fluid, and time to readiness for discharge.

**Results:**

At the 6th hour, the QoR-40 was higher in the OFA than in the MMA group (respective median [IQR] values: 180 [173–195] vs. 185 [173–191], *p* < 0.0001), but no longer difference was found at the 24th hour (median values = 191 in both groups). OFA also significantly reduced postoperative pain and morphine consumption (20 mg [1–21] vs. 10 mg [1–11], *p* = 0.005), as well as time to oral fluid tolerance (238 [151–346] vs. 175 min [98–275], *p* = 0.022), and readiness for discharge (505 [439–626] vs. 444 min [356–529], *p* = 0.001), but did not influence time to ambulate.

**Conclusion:**

While regional anaesthesia achieved most of the intraoperative analgesia, avoiding intraoperative opioids with the help of this OFA protocol was able to improve several sensible parameters of postoperative functional recovery, thus improving our knowledge on the OFA effects.

**Clinical trial number:**

Registration number NCT04285255.

## Key points

- Combined Opioid free and loco-regional anaesthesia provides enhanced early recovery
- Opioid free reduces postoperative pain intensity, and opioid consumption.
- Opioids free anesthesia allows early tolerance of oral fluid intake and expedites readiness for discharge than multimodal analgesia.

## Background

Morbid obesity is the leading cause to premature death worldwide. Bariatric surgery remains the only proven effective and durable therapy thus far. However, safety concerns; poor functional outcome including food intolerance, pain and downtime represent the main deterrents for many patients to undergo bariatric surgery. The simplicity and success of laparoscopic sleeve gastrectomy have encouraged more patients to undergo bariatric surgery and the focus has shifted on improving acceptance of the procedure including shifting it to the ambulatory care setting [1, 2].

ERAS represents the best comprehensive quality improvement program offered with laparoscopic sleeve gastrectomy to improve accessibility to the procedure. While many anaesthetic considerations in ERAS have been well established including the multimodal analgesic approach, there is still debate as to whether opioid-free anaesthesia may offer additional benefit over multimodal analgesia to achieve the goals of ERAS [3].

Opioids increase nausea, vomiting, and the likelihood of respiratory depression in the peri-operative period, especially in morbidly obese patients [4]. Furthermore, opioids may delay patient meeting extubation criteria and cause hypoventilation, muscle fatigue, ileus, and urinary retention [5].

Opioid free anaesthesia limits opioid use during the peri-operative period through the use of loco-regional anaesthesia to block nociception [6]; and use of non opioid agents with proven analgesic efficacy like dexmedetomidine, an α2 agonist [7, 8], lidocaine [9, 10] or ketamine, a NMDA receptor blocker [11, 12]. Several reviews compared opioid anaesthesia to opioid-free anaesthesia in the bariatric population but failed to provide reliable or valid conclusions due to lack of study power or variability in drug therapy or measured outcome [13, 14].

Our study, a single-blinded randomized controlled trial, compared the early postoperative quality of recovery between multimodal analgesia and opioid-free anaesthesia under ERAS in adult patients undergoing laparoscopic sleeve gastrectomy using the quality of recovery 40 (QoR-40) questionnaire at 6 and 24 h [15]. Postoperative readiness to discharge, time to ambulate, and tolerate oral fluid, degree of pain control and subsequent opioid consumption were also studied as secondary outcomes.

## Methods

The study was conducted at Almashfa Medical Center in Alkhobar Saudi Arabia between March and August 2020 (ClinicalTrials.gov: NCT04285255; registered Study Chair: Mohamed Ibrahim; registration date: February 2020). Approval was provided by Almashfa ethics committee (Chairperson Dr. Mohamed Ramadan) on January 20, 2020, under the number 1/1–2020. The trial was registered before patient enrollment. A hundred and eight patients provided written informed consent to enroll in the trial before undergoing laparoscopic sleeve gastrectomy. This manuscript adheres to the applicable Equator guidelines.

### Study design

**Fig. 1:**
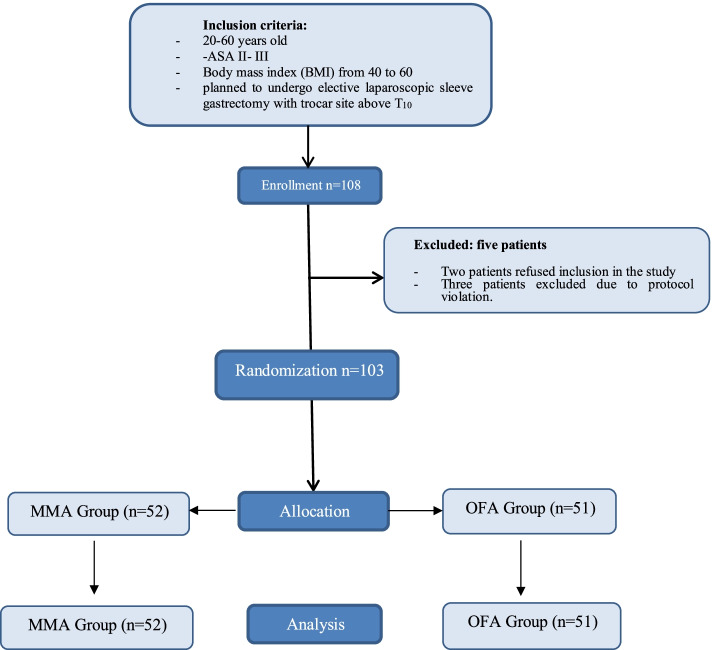
Study Flow chart showing inclusion, enrollment, randomization, allocation, and analysis

A single-blinded randomized control trial was designed using the sealed envelope method and computer-generated random numbers were kept with a pharmacist. The original random allocation sequence was locked and a copy was used instead. A non-participant nurse anaesthetist prepared syringes with the study medications (propofol, ketamine, dexmedetomidine, lidocaine, and bupivacaine for oblique subcostal transversus abdominis plane (OSTAP) block and placed them into opaque envelopes according to the allocation orders (Fig. 1).

The patient, surgical team, operating theatre staff, PACU, and surgical ward nurses were blinded to both groups. The anesthesiologist or principal investigator was the only individual aware of patient allocation and administered anaesthesia to all study patients. Sealed envelopes were labelled as multimodal analgesia (MMA) or Opioid free anaesthesia (OFA) and envelopes were only opened upon patient entry into the operating theatre.

Eligible patients included adult patients between the age of 18 and 65; Body mass index between 40 and 60; American Society of anaesthesia (ASA) class II or III; planned for elective laparoscopic.

sleeve gastrectomy through trocars positioned at or above the umbilicus (T10 dermatome). Exclusion criteria included preoperative chronic use of opioid or NSAID; allergy to bupivacaine; local skin infection at the injection site of OSTAP (oblique subcostal transverse abdominis plane block); liver or renal insufficiency; psychiatric, or neurological disease; prior open abdominal surgery above T10 dermatome; patients converted to open surgery; and patients expected to be subjected to more tissue trauma.

A team of a single anaesthesiologist and surgeon standardized the preoperative workup, patient preparation; and arranged appropriate referrals to medical services for comorbidity optimization. Patients received preoperative teaching on how to rate his pain on defined numerical rating scale (NRS) with 0 = no pain and 10 is the worst imaginable pain at the specified time verbally or written. All operations were performed using a standardized technique. An investigator blinded to both study groups was trained to interview patients and fill the quality of recovery 40 questionnaires (QoR-40) at 6 and 24 h postoperatively.

### Enhanced recovery and anaesthesia protocol

A dedicated bariatric coordinator provided preoperative teaching to patients and caregivers about the QoR-40 questionnaire and total plan of care: Preoperative oral fluid intake was encouraged, and patients were instructed to drink a 100 mg carbohydrate (CHO) load at midnight, followed by a 50 mg CHO load 2 h before surgery. Patients were premedicated with intravenous (IV) midazolam 25 μg.kg^− 1^ in the preoperative holding area. Normothermia was maintained, and sequential pneumatic compression was applied before the start of surgery.

Following maximal pre-oxygenation (end-tidal oxygen > 90%), general anaesthesia was induced using IV propofol 2 mg. kg^− 1^ and fentanyl 1 μg. kg^− 1^ in the MMA group while patients in the OFA group were premedicated with IV dexmedetomidine 0.1 μg.kg^− 1^ in 100 ml normal saline over 10 min then induced with propofol (2 mg. kg^− 1^) -ketamine (0.5 mg. kg^− 1^) mixture and maintained on dexmedetomidine 0.5 μg. kg^− 1^.h^− 1^, ketamine 0.5 mg.kg^− 1^.h^− 1^, and lidocaine 1 mg. kg^− 1^.h^− 1^ were prepared in 50 ml normal saline to run at a rate of 50 ml.h^− 1^. Cisatracurium (0.15 mg.kg^− 1^) was used in both groups for muscle relaxation. Dosage was based on the ideal body weight using the Devine formula [16].

All Patients underwent endotracheal intubation and placement of a 36 French gastric calibration tube under video laryngoscopic guidance. Ultrasound-guided bilateral oblique subcostal transverse abdominis plane (OSTAP) block was performed in all patients using 40 ml of 0.25% bupivacaine hydrochloride (Marcaine, Astra Zeneca UK) following intubation. Anaesthesia was maintained using Sevoflurane to 1.5–2.0 minimum alveolar concentration in air/oxygen with fractional inspired oxygen of 0.6, and a bispectral index (BIS) range between 40 and 60 in all patients. Standard ASA monitoring of patients included ECG, heart rate, pulse oximetry, non-invasive blood pressure, end tidal Co2 (Etco2) and core temperature.

The Ventilator was set to maintain normocapnia of 35–45 mmHg and Spo2 between 94 and 100%. All patients received parecoxib 40 mg IV after induction, and 1 g of paracetamol IV approximately 15 min prior to extubation. Furthermore, patients received dual intravenous antiemetic therapy; 8 mg of ondansetron, and 8 mg of dexamethasone.

Intraoperative hemodynamic parameters (MAP and heart rate) were recorded at 5-min intervals. Baseline values were taken 5 min after induction and a 15% rise in the MAP or HR prompted the administration of a 20 μg fentanyl bolus in the MMA group versus a 10 mg bolus of labetalol in the OFA group. Events number of MAP reduction (more than 20% from baseline) and HR (less than 45 bpm) were recorded and managed by vasopressor or atropine respectively. Duration of surgery was defined as the time from the first incision to the completed wound dressing. After surgery, the reversal of neuromuscular blockade was administered to achieve a TOF of 0.9. Extubation time was defined as the time between the end of surgery and endotracheal extubation.

In the PACU, nurses blinded to the two groups administered 15 mg of pethidine if the NRS was between 4 and 6 (moderate pain) versus 30 mg if the NRS was greater than 7 (severe pain). The pain was assessed at 5-min intervals until pain relief was achieved (NRS ≤3). Total pethidine dose in the PACU was recorded. The level of sedation was assessed 15 min after arrival to the PACU according to the Ramsay score [17]. Low saturation (< 94%), obstructed breathing, shivering or feeling cold, and duration of PACU stay were also recorded. Patients were discharged from the PACU if they achieved a modified Aldrete score of ≥9 [18].

In the ward, all patients received 1 g of IV paracetamol 6 hourly, and 40 mg IV parecoxib 12 hourly. Level of pain was measured using NRS on arrival to the ward at 0 h, and at 2, 4, 6, 12, and 24 h postoperatively. An intravenous infusion of 25 mg of pethidine over 15 min was administered if NRS > 3, and the pain was re-assessed at 15 min intervals. An additional 10 mg intravenous infusion of pethidine was given as needed till the NRS score dropped below 4. Four mg of Intravenous ondansetron and or 10 mg of metoclopramide were administered to treat nausea or vomiting.

Patients were encouraged to ambulate, start oral ice chips, and void within 2 h from arrival to the ward. The timing of the first rescue analgesic dose or need for antiemetic therapy in the ward were recorded as well as further doses during the 24-h stay.

#### Primary outcome

A blinded investigator trained on the QoR-40 interviewed all patients and completed the form at 6 and 24 h from patient arrival to the ward. A transculturally validated arabic version of the questionnaire was used by the investigator in the interview. Twelve questions measured the comfort state; 9 questions measured the emotional state; 7 questions measured the psychological state; 5 questions measured the physical independence and 7 questions measured the level of pain. Each question received a score of 1 to 5 with a worst possible score of 40 and a best possible score of 200 [19].

#### Secondary outcome

Time to first independent ambulation, time to tolerate oral fluids, and time to readiness for discharge were measured according to the modified postoperative discharge scoring system (PADSS) [20]. The Blinded investigator-assessed patient readiness for discharge according to PADSS on an hourly basis and documented the time to achieve discharge eligibility in minutes. Patients were considered eligible for discharge if they achieved a total score ≥ 9 on the condition that the vital signs parameter score was not less than 2, and none of the other five parameters scored a zero.

Numerical rating scale (NRS) in the PACU, and on arrival to the ward at 0 h then at 2, 4, 6, 12, and 24 h postoperatively were recorded. Total pethidine consumption, calculated as an equivalent oral morphine dose, during the 24 h, in the recovery unit (PACU) and at time of first rescue analgesic dose in the ward were recorded as well as the proportion of events with NRS >4.

Intraoperative hemodynamic parameters, surgical time, extubation time, PACU stay, and postoperative nausea and vomiting in the PACU, and in the ward were also documented.

### Statistical analysis

Statistical analysis was done using IBM-SPSS 20 software (IBM Corp., Armonk, N.Y., USA). Prior published studies on heterogeneous patients undergoing cardiac, general, and neurosurgical procedures have suggested a 3.2% difference in the QoR-40 score as clinically significant which we have used to calculate our sample size [21]. Adoption of ERAS in MMA and the use of OSTAP block have already improved the quality of recovery in laparoscopic and bariatric surgery to above the 90th percentile in almost all patients. Nowadays, most of the work focuses on improvement in the remaining 10% of the score. A difference of 1.5% in the score will translate to a 15% improvement in the non-achieved score and would probably be clinically significant if it results in change in the time to discharge, a target of ERAS. A sample size of 43 patients in each arm had a 90% power to detect the 3.2% difference in the mean score with a 3.5% standard deviation at an alpha of 0.05 using a two-sided two-sample equal-variance t-test. Fifty-four patients were recruited in each arm assuming a 20% drop-out rate.

The normality of the distribution of each of the continuous data was examined by the Kolmogorov-Smirnov test and quantile–quantile plot. Normally distributed continuous variables were presented as mean ± SD; and were univariately compared using a two-sample Student’s t test. When continuous data were not normally distributed: the median, the interquartile range were presented and were compared using the Mann–Whitney test. The frequency or percentage was used to describe categorical data, and Fisher exact was the preferred test to compare the two groups whenever appropriate followed by the Pearson’s chi-square test.

Number of events (number of events over total number of measurements) was used to analyze reduction in MAP (more than 20% reduction from baseline), HR (less than 45 bpm) and NRS (more than 3) repeated measurements.

## Results

One hundred and eight patients were allocated to both groups. Two patients refused inclusion in the study; three patients were excluded due to protocol violation (two patients received an out of protocol different postoperative analgesia by a new on-call physician unaware of the study protocol, and the third patient received sedation in the PACU due to severe agitation. One hundred and three patients were randomly allocated to the MMA or OFA groups. Patient characteristics were similar between the two groups (Table 1).

**Table 1:** Clinical characteristics. Mean ± SD, standard deviation; M, male: F, female; BMI, body mass index; IQR, Interquartile range, Times are shown in minutes

	Group I (MMA) (***N*** = 52)	Group II (OFA) (***N*** = 51)	***P*** value#
**Age (yr.)**^a^	32.0 [23.0–39.0]	30.0 [22.0–36.0]	0.242
**Sex (M/F)**^b^	21/31	19/32	0.745
**ASA II**^b^	35	29	0.275
**ASA III**^b^	17	22	
**BMI (kg/m**^**2**^**)**^a^	44.0 [42.0–45.0]	45.0 [43.0–46.0]	0.062
**Duration of surgery (min)**^c^	43.0 ± 9.86	40.5 ± 9.04	0.172
**Duration of anaesthesia (min)**^c^	64.8 ± 9.19	63.7 ± 8.61	0.524

-Values are given as mean ± standard deviation and median [IQR]

#*P* value<0.05 significant

^a^Mann–Whitney test

^b^Fisher’s exact test

^c^Student’s t-test

### Quality of recovery

All patients answered the QoR-40 questionnaire without difficulty. The estimated difference in mean was used to compare the QoR-40 scores in the pre-study protocol. The total estimated difference in the mean QoR-40 score was −4.15, 95% CI (−5.78 to −2.5) with a *P* value <0.0001 at 6 h; and was 0.738, 95% CI (− 0.8 to 2.28), with a *P* value = 0.345 at 24 h. However, we discovered later that the QoR-40 score followed a non- Gausian distribution. Hence, we used the Mann-Whitney’s test to compare the median difference in the QoR-40 scores (Table 2). Both parametric and non-parametric tests gave the same level of significance.

**Table 2:** The dimensions of the postoperative QoR-40 at 6 and 24 hours

	Group I (MMA) (***N*** = 52)	Group II (OFA) (***N*** = 51)	***P*** value#
**QoR-40 at 6 h**			
**Comfort (60)**	52.0 [44.0–58.0]	53.0 [45.0–58.0]	0.004^#^
**Emotion (45)**	42.0 [40.0–51.0]	44.0 [36.0–45.0]	<0.0001^#^
**Physical independence (25)**	23.0 [22.0–25.0]	23.0 [22.0–23.0]	0.004^#^
**Psychological support (35)**	34.0 [33.0–35.0]	34.0 [33.0–35.0]	0.016^#^
**Pain (35)**	30.0 [28.0–33.0]	32.0 [28.0–32.0]	<0.0001^#^
Total score (200)*	180 [173–195]	185 [173–191]	<0.0001^#^
**QoR-40 at 24 h**			
**Comfort (60)**	56.0 [50.0–60.0]	56.0 [48.0–60.0]	0.198
**Emotion (45)**	44.0 [41.0–45.0]	44.0 [37.0–45.0]	0.810
**Physical independence (25)**	24.0 [23.0–25.0]	24.0 [21.0–24.0]	0.310
**Psychological support (35)**	35.0 [34.0–35.0]	35.0 [33.0–35.0]	0.275
**Pain (35)**	32.0 [29.0–35.0]	32.0 [30.0–34.0]	0.534
**Total score (200)**	191 [181–198]	191 [178–198]	0.479

The values are the median [Range]

Analysis by Mann-Whitney’s test

#*P* value < 0.025 significant

Significance was preserved after correcting the type-I error by the Bonferroni’s correction for two measurements (significance threshold at 0.025)”.

### Readiness for discharge

The OFA group tolerated oral fluid earlier and quicker readiness for discharge than MMA group, *P*-value = 0.022 and 0.001 respectively but did not influence time to ambulate (*p* = 0.169) (Table 3).

**Table 3:** Comparison of analgesic efficacy and recovery criteria between both groups

	Group I (MMA) (***N*** = 52)	Group II (OFA) (***N*** = 51)	***P*** value
PACU morphine analgesia (mg)	10 [10–20]	10 [0–10]	0.024
Total postoperative morphine consumption (mg) [PACU and Ward]	20 [10–30]	10 [10–20]	0.005
Number (%) of patients did not receive opioids in PACU	10 (19.2)	18 (35.3)	0.068
Number (%) of patients did not receive opioids in ward	17 (32.7)	24 (47.1)	0.136
Number (%) of patients did not receive opioids in PACU or ward	7 (13.5)	7 (13.7)	0.969
Median proportion of events NRS > 3	0.58 [0.41–0.66]	0.50 [0.16–0.66]	0.008
Extubation time (min)	5.50 [4.0–9.5]	9.0 [9.0–11.0]	0.0002
PACU stay (min)	20.0 [20.0–25.0]	30.0 [25.0–35.0]	<0.0001
Time of first rescue analgesia	120.0 [69.0–152.0]	228.0 [122.5–229.0]	<0.0001
Ramsay Sedation score in the PACU	3.0 [3.0–3.0]	4.0 [3.0–4.0]	<0.0001
Time to ambulate (min)	169 [122.5–216.5]	150 [107.5–195.5]	0.190
Time to oral fluid tolerance (min)	238 [150.5–346.0]	175 [97.5–274.5]	0.022
Time to readiness for home discharge (min)	504.5 [439–625.5]	444.0 [356–529]	0.0009

Analysis between groups was done using Mann-Whitney test

Values are given as median, [IQR] -*P* value < 0.05 is considered significant

### Pain assessment

**Fig. 2:**
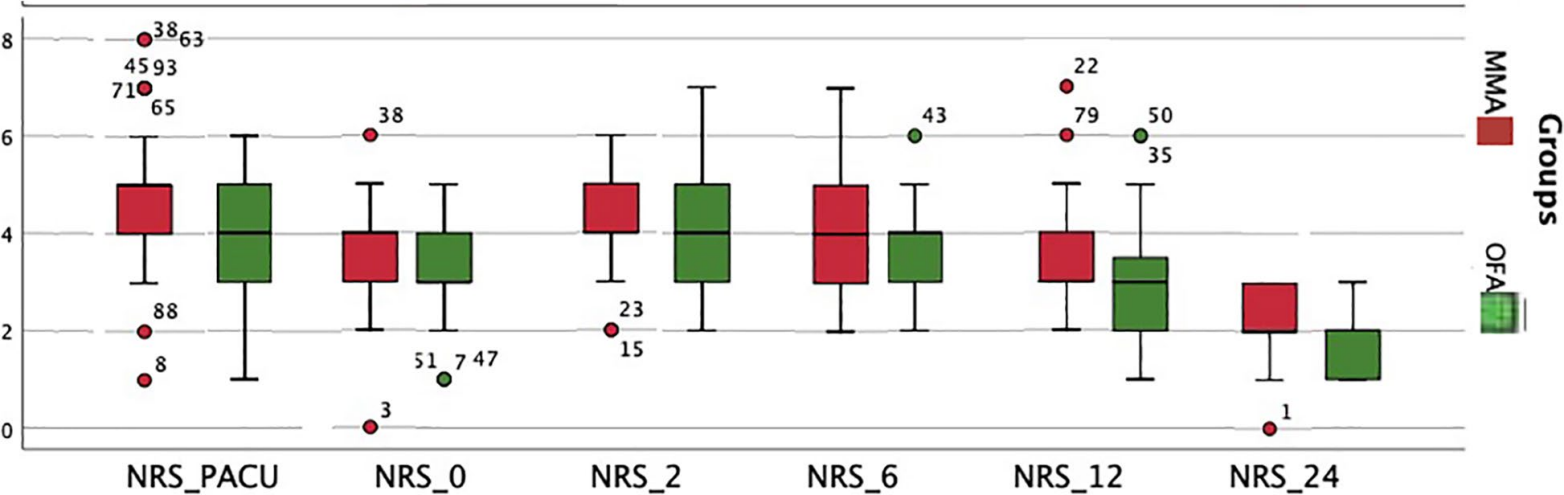
Numerical rating scale (NRS) at PACU and 24 h. Boxplot distribution of the NRS over time. Median is represented by a dark horizontal line, interquartile range by the upper and lower limits of the box, extreme values are represented by whiskers, outliers are represented by circles with numbers representing the case order

The median and interquartile range for the NRS scores in the OFA group was statistically lower than MMA group at PACU, and at 2 and 6 h (Fig. 2). Furthermore, the proportion of events median value with NRS > 3 was 0.50 in the OFA group vs. 0.58 in the MMA group, *P* = 0.008 (Table 3).

### Opioid consumption

The parameters of opioid consumption (in PACU and total 24-h) were lower in the OFA group *P* = 0.005 and 0.024 respectively (Table 3).

In the MMA group, sixteen (30.7%) patients required rescue fentanyl for an MAP elevation with a mean dose of 11.83 ± 20.79 μg; Eight patients (15.3%) required a single rescue dose; Seven patients (13.5%) required two doses; and only one patient required a third rescue dose. The median value of MAP reduction events was 0.28 in the OFA group vs. 0.1 in the MMA group, *P* = 0.002. Only 3 patients in OFA group had severe bradycardia (HR less than 45 bpm) and treated promptly with atropine.

**Fig. 3:**
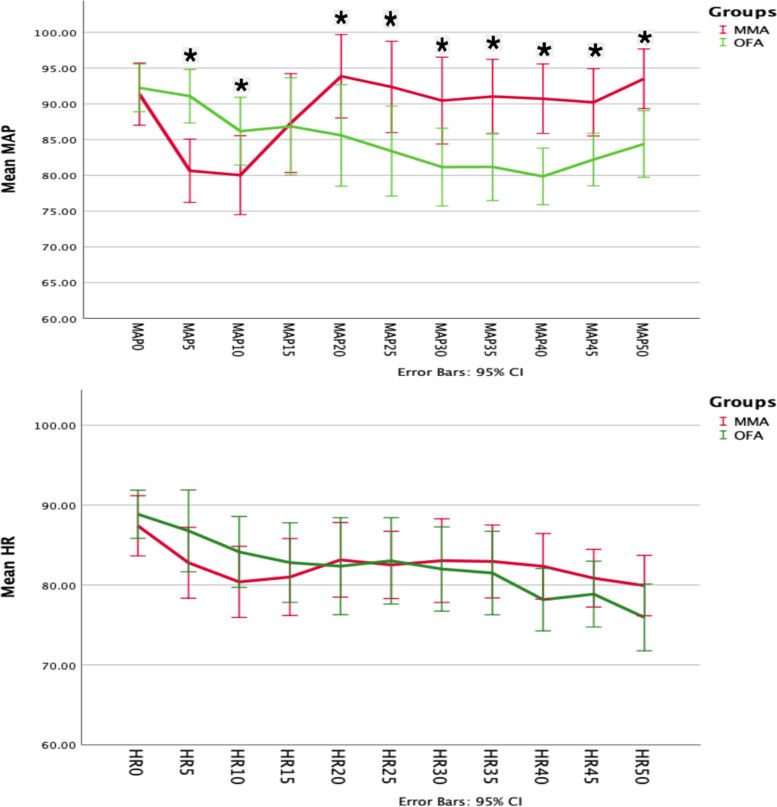
Intraoperative hemodynamics changes. *Statistically significant

None of the patients in the OFA group needed labetalol for an elevation in the HR or MAP. Hemodynamics (HR and MAP) changes during surgery are shown in Fig. 3. Extubation time was 6.85 ± 3.81 min in the MMA group versus 9.24 ± 3.29 min in the OFA group, *P* = 0.001.

Eighteen patients (35.3%) in the OFA group experienced postoperative nausea or vomiting (PONV) versus 24(46.2%) in the MMA group, *P* = 0.262. Nine patients (17.6%)in the OFA group had hallucinations versus none in the MMA group. There were no observed adverse events related to the OSTAP block, and no symptoms or signs of systemic toxicity were associated with the use of local anesthetic agents.

## Discussion

In our study, OFA enhanced the total quality of recovery score and the individual scores of its five dimensions at 6 h when compared to MMA using the validated QoR-40 questionnaire. Such enhancement disappeared at 24 h. In essence, our study showed MMA had slower recovery when compared to OFA making the latter more suited for ERAS. OSTAP block under ERAS resulted in all patients achieving the 90th percentile. A change in the remaining 10 percentile is what any study should target within ERAS. Our study showed a 2.1% absolute difference which translates to a 21% improvement in the remaining 10 percentiles which is considered clinically relevant because it resulted in early tolerance of oral fluid intake and expedited discharge in the OFA group. Mulier et al. [22] in a randomized controlled trial conducted on 45 patients found the QoR-40 to be better in the opioid-free anaesthesia when compared to the opioid anaesthesia even at 24 h (*p* < 0.001). We feel the use of a loco-regional block in our study and lack of maintenance opioid infusion improved the score in our MMA group by 27% over the opioid group in the Mulier study, and hence reduced the difference between the two groups in our study to a non-significant level at 24 h.

Most studies have compared full opioid versus opioid-free anaesthesia [23–28]. Total opioid anaesthesia is falling out of favour and is rarely practiced since it is unanimously agreed that reducing opioid is beneficial. No studies have compared reduced opioid under multimodal analgesia versus opioid-free anaesthesia combined with loco-regional anaesthesia.

The low intraoperative opioid dose in our MMA group and in both groups postoperatively were deliberate. We tested OFA in a non-conventional way, as the control group had few intraoperative opioids, as all patients were covered by the intraoperative TAP block.

In a meta-analysis, Fletcher and Martinez showed that intraoperative administration of large doses of opioids caused increased pain perception, and postoperative opioid consumption [29]. The opioid paradox or opioid hyperalgesia is a state in which increased opioid dosage increases pain receptor sensitization, especially following short-acting opioids [13, 30]. Furthermore, opioid tolerance plays a role in increasing postoperative demand [31–33].

The improved score in the pain dimension of the QoR-40 at 6 h in the OFA group was further supported by a reduced mean NRS score at 6 h in the same group. The combination of Dexmedetomidine [8], lidocaine [9], and ketamine [12] reduced the number of events with an NRS score > 4; resulting in reduced opioid consumption in the PACU and ward. Even though fewer patients in the OFA group needed opioid analgesia in the PACU or ward, the difference failed to reach statistical significance (Fig. 2).

A retrospective study showed positive correlation between postoperative morphine consumption and the pain score, and just implementing ERAS resulted in a 41.8% reduction in the morphine equivalent dose, *P* < 0.001 [34].

Zeltsman and colleagues [13] found more opioid consumption in the OFA (23.25 mg) vs. 9.79 mg in OA, *P* = 0.03; a longer time 255.3 min to achieve an Aldrete score > 9 in OFA versus 135.42 min in OA, *P* = 0.03; and 70% of OFA patients needed antiemetic rescue doses vs. 25% of OA patients, *P* = 0.08. It is noteworthy that the pain score in both groups and PACU stay was much higher when compared to other studies in the literature. Though the authors cautioned against drawing a conclusion due to the small sample size and the possibility of random error, their study highlights the importance of not adopting OFA outside of an ERAS protocol.

Ziemann-Gimmel et al. [23] showed no difference in postoperative opioid consumption between OFA and OA. Yet, the OFA group had a statistically significant reduction in nausea (*p* = 0.02). The use of a lower ketamine dose (0.5 mg.kg^− 1^) at induction, as well as not having any form of regional block may explain the lack of difference in opioid consumption. The reduction in nausea in their OFA group was similar to what we have seen though it did not reach significance in our study. Furthermore, the significant absolute reduction in the proportion of patients suffering from nausea compared to many studies including ours might be explained by the pre-emptive use of a scopolamine transdermal patch plus triple prophylaxes with dexamethasone, ondansetron, and rescue doses of droperidol or promethazine in the PACU.

Several trials implementing the ERAS pathway in bariatric surgery concluded its efficacy in reducing the length of hospital stay, peri-operative opioid consumption, readmission rate, and incidence of PONV [35–40]. Patients in the OFA group were able to tolerate fluids earlier, had less nausea or vomiting, and potentially reduced their stay by 18%, a desired outcome of ERAS in the era of managed care and capitation.

Use of Ketamine in short duration surgery in our patients may explain the prolonged extubation time, longer duration, and higher sedation score in the PACU for the OFA patients but it had no clinical relevance since the PACU stay was much shorter than most reported studies in the literature. There were 9 cases of mild hallucination (17.6%) in the OFA group, a side effect reported in one trial using nearly the same ketamine dose [23]. Hallucinations were short-lived, self-aborted in the PACU, and were not remembered in the ward. Hemodynamics stability (MAP) was better under OFA and none of our patients experienced hypoxemia in the PACU.

Finally, this is the first high powered randomized trial that shows OFA can be more suited for ERAS in bariatric surgery.

We realized from a pilot study performed on ten patients that the first 6 h were more relevant in sleeve gastrectomy done under ERAS so we decided to add a measurement of the quality of recovery at 6 h which proved later to be valuable. Inability to compare the length of stay between both groups is one limitation of the study since the health authority in the country forbids same-day discharge in bariatric surgery. We believe our study will encourage change to this policy. To overcome this limitation, we measured the time to readiness for discharge based on PADSS as a substitute. Implementing ERAS has been proven to reduce hospital stay [32]. Our study showed adding OFA expedited readiness for discharge by an absolute 22% in addition to the improvement in the quality of recovery as discussed earlier.

A second limitation or challenge occurred during blinding and provision of the intervention by the principal investigator. That was overcome by having an independent second investigator collect and analyze the data independently.

## Conclusion

Opioid free anaesthesia when combined with Loco-regional anaesthesia is better suited to satisfy the goals of ERAS. It provides early improvement of the quality of recovery; improves postoperative pain; and reduces opioid consumption over multimodal analgesia. A larger multi-centre randomized controlled trial is needed to standardize the optimal anaesthetic and surgical considerations in ERAS in bariatric surgery.

## Data Availability

The data set used and/or analyzed during the current study are submitted in a separate file. Data can be made available from the corresponding author upon request at any stage.

## Abbreviations

ASA: American Society of Anesthesiologists
BIS: Bispectral index
BMI: Body Mass Index
ERAS: Enhanced Recovery after Surgery
Etco2: End tidal Co2
IV: Intravenous
IQR: Interquartile range
MAP: Mean arterial pressure
MMA: Multimodal analgesia
NMDA: N-methyl d aspartate
NSAID: Non-steroidal anti-inflammatory drugs
NRS: Numerical rating scale
OFA: Opioid-free anaesthsia
OSTAP: Oblique subcostal transverse abdominis plane
PACU: Post-anaesthesia care unit
PADSS: Postoperative discharge scoring system
PONV: Postoperative Nausea and Vomiting
QOR-40: Quality of Recovery Score- 40
TOF: Train of four
